# Screening of Trace Elements in Hair of the Female Population with Different Types of Cancers in Wielkopolska Region of Poland

**DOI:** 10.1155/2014/953181

**Published:** 2014-12-15

**Authors:** Bogusław Czerny, Krzysztof Krupka, Marcin Ożarowski, Agnieszka Seremak-Mrozikiewicz

**Affiliations:** ^1^Department of General Pharmacology and Pharmacoeconomics, Pomeranian Medical University in Szczecin, Zolnierska 48, 70-204 Szczecin, Poland; ^2^Department of Pharmacology and Phytochemistry, Institute of Natural Fibers and Medicinal Plants, Wojska Polskiego 71b, 60-630 Poznan, Poland; ^3^Department of Pharmaceutical Botany and Plant Biotechnology, Poznan University of Medical Sciences, Święty Marii Magdaleny 14, 61-861 Poznan, Poland; ^4^Division of Perinatology and Women's Diseases, Poznan University of Medical Sciences, Polna 33, 60-535 Poznan, Poland; ^5^Laboratory of Molecular Biology, Poznan University of Medical Sciences, Polna 33, 60-535 Poznan, Poland

## Abstract

*Background*. Cancer constitutes a major health problem worldwide. Thus, search for reliable and practical markers of the disease process remains the key issue of the diagnostic process. *Objectives*. The study aims at linking the trace element status of an organism, assessed by hair analysis, with the occurrence of cancer diseases. *Material and Methods*. Hair samples were collected from 299 patients with cancer diseases confirmed by a histopathological test and from 100 controls. Cancer patients were divided into three groups, depending on cancer type: hormone-dependent cancer, cancer of the alimentary tract, and cancer with high glycolytic activity. Mineral element analysis of hair was performed using an atomic emission spectrophotometer with inductively coupled plasma (ICP-OES) and inductively coupled plasma mass spectrometry (ICP-MS). *Results*. Statistically significantly lower concentrations of selenium, zinc, copper, germanium and boron, iron, and magnesium were observed in the three groups of cancer patients. Disturbance in the axis glucose-insulin and changes in concentrations of heavy metals and toxic elements were also noted. *Conclusions*. It seems safe to conclude that our results confirmed usefulness of hair element analysis in screening tests for the assessment of the biomarker of various cancer diseases in a female population.

## 1. Introduction

According to the World Health Organization (WHO) [1], cancer is and will become an increasingly important factor in the global burden of disease in the decades to come. The number of new cases reported annually is expected to rise from 10 million in 2000 to 15 million in 2020. Thus, early detection may allow for early diagnosis in the symptomatic and screening in asymptomatic, but at risk, populations. Moreover, screening of seemingly healthy individuals can disclose cancer in early or precursor stages, when treatment is most effective. Therefore, there is a need for further search of appropriate screening methods and cancer markers.

In recent years, the analysis of trace elements in human tissues has attracted the attention of numerous researchers and its application continues to expand due to the role of these elements in the biochemical and physiological processes [2]. Determination of trace elements in human hair is important in biological, medical, environmental, and forensic disciplines as it represents an interesting biological matrix for various studies [3, 4]. Lately, hair has become a fundamental biological specimen, alternative to the usual blood and urine samples, as well as biopsy material, in clinical toxicology and chemistry [5–7]. Human hair has been shown to be attractive as diagnostic material due to simplicity of sampling. Moreover, hair constitutes a neutral and stable tissue material and may provide valuable information about accumulation of trace elements that are considerably more concentrated in hair than in other biological materials [8, 9]. Thus, hair analysis may provide an indirect screening test for physiological excess as well as deficiency of elements in the body. It is vital to note, as was summarized by Rȩbacz-Maron et al. [5], that the content of chemical elements in hair is determined, among others, by diet, sex, age, race, individual demand of each organism, socioeconomic conditions, the content of chemical elements in drinking water, geographical location, and environmental pollution. The main advantage of this method is that it enables monitoring the changes in trace element status in the body over a long period of time, much longer than in case of blood samples. Nowadays, clinical research indicates that levels of certain trace elements in hair (particularly potentially toxic elements) are highly correlated with pathological disorders [10]. A growing amount of data supports the theory that biochemical analysis of trace elements in hair may be useful in identifying the possible risk of cancer development or progression as simple biomarkers without the need for an invasive biopsy. Silva et al. [2] demonstrated that investigation of trace elements in cancer tissues may be regarded as tumor biomarkers and prognostic factors in breast cancer. Other authors showed also that alterations in trace elements in plasma and cancer tissues were observed in patients with, for example, colorectal cancer [11, 12], malignant breast tissues [13], malignant prostate [14], cancerous endometrial and ovarian tissues [14], and head and neck cancers [15]. Also, several studies have focused on the relationship between scalp hair trace element levels and cancer in patients from various geographical locations, that is, Turkey (Anatolia) [16], India (Malwa region, Punjab) [17], Pakistan (Rawalpindi district; Jamshoro) [6, 18–21], Iran (Tehran) [22], Italy (Modena region) [23], and China (Guangdong Provence) [24, 25] but not Poland. The role of trace elements in the development or inhibition of cancer remains to be fully elucidated. Moreover, that field has not been extensively studied in cancer patients in Poland. The main objective of our study was to assess the concentrations of trace elements in hair of cancer patients hospitalized in clinical hospitals (Pomeranian Medical University in Szczecin) by atomic emission spectrometer with inductively coupled plasma (ICP-OES) and inductively coupled plasma mass spectrometry (ICP-MS) by NZOZ Biomol-Med Sp. z o.o. (Łódź, Poland).

## 2. Material and Methods

### 2.1. Patient Groups

A total of 399 Polish women were included in the study, both for the cancer and control groups. Individuals using supplementation with trace elements and vitamins during the last three months preceding the study were excluded. All patients had been histopathologically tested for the disease and were randomly selected from the clinical hospitals at the Pomeranian Medical University (Szczecin, Poland). Cancer patients (aged 35–60 years) were divided into three groups. Group 1 (H) was comprised of 98 females with hormone-dependent cancers, such as breast and ovarian carcinomas. Group 2 (HG) consisted of 101 patients with cancers characterized by high glycolytic activity system, such as Hodgkin's lymphoma, leukemia, non-Hodgkin lymphoma, melanoma, and brain tumor. Group 3 (D) was composed of 100 women suffering from digestive tract cancers. Healthy volunteers (*n* = 100, aged 25–40 years) were recruited as controls (C). The study was approved by the local ethics committee (Pomeranian Medical University).

### 2.2. Sample Collection, Preparation, and Analysis of Hair Element Composition

The 3-4 cm hair samples were obtained from the head, according to widely accepted standards, that is, hair without perming or coloring, cut from the back of the head (from a few places), close to the skin. The weight of a hair sample ranged between 300 and 400 g. The samples were washed in solutions of nonionic detergents and then dried to constant mass.

An accurately weighed portion (0.3 g) of the hair sample was placed in flasks with 25 mL capacity. 5 mL of a freshly prepared mixture of concentrated 65% HNO_3_-H_2_O_2_ (2 : 1, v/v) was added to each flask and heated at 80°C for 10 min in accordance with the method described above [16]. Final solutions were made up to 10 mL with 2 mol/L HNO_3_. The samples were mineralized in a closed system in an ETOS microwave station (Mileston). Triplicate scalp hair samples of each cancer patient and healthy participants were treated as described above. The analysis of the hair element composition was made with an atomic emission spectrometer with inductively coupled plasma (ICP-OES), Optima 5300 DV (Perkin Elmer 2300 D), and inductively coupled plasma mass spectrometry (ICP-MS; Perkin Elmer DRC II). The source materials for comparison were reference materials compliant with the standard. The apparatus was calibrated using standard solutions. The calibration curve was drawn automatically by the computer coupled with the apparatus.

Analytical figures such as calibration curve for each trace elements, the linear correlation coefficient for calibration curves (*r*), coefficient of variance (SD_2_), detection limits, certified reference material, and sample volume used in this study were summarized in Table 1.

All reagents were purchased from Sigma-Aldrich (Poland) and Merck (Poland). The content of 23 nutritional elements, that is, arsenic (As), Barium (Ba), boron (B), cadmium (Cd), calcium (Ca), cobalt (Co), copper (Cu), chromium (Cr), germanium (Ge), iodine (I), iron (Fe), lithium (Li), magnesium (Mg), manganese (Mn), molybdenum (Mo), nickel (Ni), potassium (K), selenium (Se), silicon (Si), sodium (Na), strontium (Sr), sulfur (S), tin (Sn), vanadium (V), and zinc (Zn), and of 6 toxic elements, aluminum (Al), lead (Pb), and mercury (Hg) was determined in the collected samples. The results were assumed to be the so-called “element status.”

### 2.3. Statistical Analysis

Statistical analysis of the results was performed using the software package Statistica 7.1. All values were expressed as means ± SEM and results were expressed as micrograms per gram. The statistical comparison of the results was carried out using the Kruskal-Wallis test (nonparametric several independent samples test) to evaluate differences between the three cancer groups and healthy controls and also by the Mann-Whitney test* U*, setting *P* < 0.05 as the limit of significance.

## 3. Results 

Mean concentrations with standard deviations (± SEM) for each analyzed chemical element in hair of cancer patients and healthy participants are presented in Table 2. The results indicate that concentrations of essential trace and toxic elements in the biological samples of cancer patients were altered.

### 3.1. Hormone-Dependent Cancer Group (H)

Statistical analysis revealed that, in the group of patients with hormone-dependent cancers, levels of 7 elements (K > Al > Hg > Ca> Na > Mo = V) were higher as compared to the control group, whereas hair of cancer patients contained significantly lower levels of 9 elements (B > Cu > Fe > Se > Ge > Mg > Cr > Mn > Zn) (Table 2) and significant changes were observed in calcium, sodium, magnesium, iron, and copper (*P* < 0.0001), potassium (*P* < 0.0002), zinc (*P* < 0.0351), manganese (*P* < 0.0043), selenium and chromium (*P* < 0.0001), molybdenum (*P* < 0.0011), vanadium (*P* < 0.0036), and germanium (*P* < 0.0001) concentrations, as compared to the control group. Significant differences of the concentrations were also observed in case of toxic metals: aluminum (*P* < 0.0007) and mercury and boron (*P* < 0.0001) (Table 2). The level of K in this cancer group was calculated to be 2.13 times higher than in controls. In contrast, decreased level of B (by 58.1%) was noted. Moreover, statistically significant differences were visible in the ratio of the concentration of the tested elements in this group as compared to the control group: Ca/P (*P* < 0.0001); Na/K (*P* < 0.0001); Zn/Cu (*P* < 0.0001); Ca/Mg (*P* < 0.0001); Ca/Na (*P* < 0.0339); and Ca/Pb (*P* < 0.0001). Moreover, the results of the tests were run against the values obtained in the other groups of cancer patients, that is, with digestive system cancer (D) and with high glycolytic activity cancers (HG). In this group, copper concentration was lower in comparison with both D (*P* < 0.0001) and HG (*P* < 0.0437) groups. As compared to the other groups of cancer patients, lower concentrations of iron (*P* < 0.0033) and zinc (*P* < 0.0001) were observed, although this value is statistically significant only in comparison to the D group. It was also noted that the ratio of Zn/Cu concentration was the highest in this group, both with respect to the HG group (*P* < 0.0032) and the D group (*P* < 0.0001).

### 3.2. High Glycolysis Cancer Group (HG)

Statistical test revealed that, in the HG group (containing brain tumors, lymphoid system cancers, and leukemia), considerable changes were observed in concentrations of 9 nutritional elements (increased level for Al > Cd > K > Hg > Na > Pb > Mo = V > P) and 8 elements (decreased level for Mg > Ge > Se > B > Cu > Ca > Fe > Zn), with statistical significance for calcium, phosphorus, and potassium (*P* < 0.0001), sodium (*P* < 0.001), zinc (*P* < 0.0096), and magnesium, iron, copper, selenium, molybdenum, vanadium, and germanium (resp., *P* < 0.0001) as compared to the control group. In case of toxic metals, the data indicate statistically significant differences in concentrations of aluminum (*P* < 0.0001), lead (*P* < 0.0359), cadmium (*P* < 0.0001), mercury (*P* < 0.0001), and boron (*P* < 0.0001) as compared to the control group. The level of Al was 2.26 times higher and the level of Mg was 64.72% lower in this cancer group in comparison to the control group. Also, statistically significant differences (in comparison to the control group) in the ratio between concentrations of the following elements: Ca/P (*P* < 0.0001); Ca/K (*P* < 0.0001); Na/K (*P* < 0.0016); Zn/Cu (*P* < 0.0001); Ca/Na (*P* < 0.0001); Ca/Pb (*P* < 0.0001); and Zn/Cd (*P* < 0.0001) were noted (Table 3). The results obtained for the HG group were compiled with the results of other cancer patients. In case of the HG group, in comparison with the D and the H groups, lower concentrations of calcium (*P* < 0.0001), magnesium (*P* < 0.0001), and germanium were detected, whereas phosphorus levels were elevated. Germanium levels were significantly lower only in relation to the D group (*P* < 0.0033). Phosphorus concentrations in the HG group reached considerably higher levels in relation to both the H group (*P* < 0.0018) and the D group (*P* < 0.0001). Moreover, the Ca/P ratio was observed to be lower than the same parameter in the other cancer groups (*P* < 0.0001).

### 3.3. Cancer of the Digestive System Group (D)

In the group of patients with cancers of the digestive system (liver, pancreas, colon, and lung cancers), substantial differences were observed in 9 nutritional elements (increased level for Al > Hg > K > Na > V > Ca > Cd > Pb > Mo) and 8 elements (decreased level for Se > Zn > Mg > B > Fe > Ge > Cu > Cr), with statistically significant values for calcium (*P* < 0.0001), sodium (*P* < 0.001), potassium, zinc, magnesium, iron, copper, and selenium (resp. *P* < 0.0001), chromium (*P* < 0.0291), molybdenum and vanadium (*P* < 0.0001), and germanium (*P* < 0.0093) as compared to the control group. Significant changes in concentrations were found (in relation to the controls) also for toxic metals: aluminum (*P* < 0.0001), lead (*P* < 0.006), cadmium (*P* < 0.0002), and mercury and boron (*P* < 0.0001). The highest and the lowest levels were noted for Al (2.6 times) and Se (2.56%), respectively. Additionally, differences in the following ratios between concentrations of the tested elements were found to be statistically significant as compared to the control group: Ca/P (*P* < 0.0001); Zn/Cu (*P* < 0.0001); Ca/Mg (*P* < 0.0001); Ca/Pb (*P* < 0.0473); and Zn/Cd (*P* < 0.0001). The results obtained for the D group were juxtaposed with the other cancer groups. In the D group the concentrations of zinc were lower than in case of other cancer groups (*P* < 0.0001). Also, selenium levels were lower too, yet the difference was statistically significant only in relation to the H group (*P* < 0.0071). The ratio of Ca/Mg concentrations in the D group was observed to be higher than in the H and the HG groups. However, the difference is of statistical importance only in relation to the HG group (*P* < 0.0001). The data on the ratios between the elements in the cancer patient groups and the control group are shown in Table 3.

## 4. Discussion 

Our study provides data on several substantial differences in the element composition of hair between cancer patients and controls and also between each group of cancer patients, that is, suffering from hormone-dependent cancers (H), cancers with the so-called high glycolytic activity (high glycemic index) (HG), and cancer of the digestive system (D). In all of the tested cancer groups, especially in the D group, statistically significant decrease in selenium levels was observed. This finding is compliant with the results of Kolachi et al. [18], who reported low levels of selenium in all three biological samples (blood, serum, and scalp hair) of liver cancer patients. Thus, it seems safe to conclude that administration of selenium may be included in the treatment of cancer of the digestive tract (chemoprevention), also due to the fact that selenium induced G2/M cell cycle arrest and apoptosis in colorectal cancer cells via Bax-dependent mitochondrial pathway [31]. Recently, selenium has been shown to induce a multitargeted cell death process in addition to ROS formation [32]. Other authors [33] also observed that dietary selenium supplementation (and green tea) is effective in suppressing colorectal oncogenesis. Most importantly, a meta-analysis of clinical studies confirmed the preventive role of selenium administration in gastrointestinal tract cancers [34]. Results of clinical trials indicate that low levels of selenium concentrations in the organism are an important factor in cancer occurrence, in particular tumors of the alimentary tract, prostate, and breasts [35–37].

Similarly, considerably lower concentrations of copper and zinc were found in hair samples of all groups of cancer patients. Also, several studies observed significant decrease in mean total concentration of Zn and Cu in hair of lung cancer patients [23], Zn in hair of the prostate cancer group [24], hair of breast cancer cases [22], and whole blood samples of breast and ovarian cancer groups of patients [21]. On the other hand, Pasha et al. [20] showed that the highest average level of Zn was found in the scalp hair of cancer patients. Zinc levels can be closely linked with its absorption in the body [38]. Particularly low levels of zinc in hair were found in the groups of alimentary tract cancers. The literature data indicate also a connection between disorders in the metabolism of zinc and the mutation of p53 gene [39, 40]. Zn deficiency was observed to cause inactivation of p53, a tumor suppressor protein, which has been associated with many cancers [41]. Moreover, epidemiologic study showed that zinc deficiency may be associated with an increased risk of various cancers and zinc supplementation is associated with decreased oxidative stress and improved immune function, which may be among the possible mechanisms for its cancer preventive activity [42]. It might be concluded that a diet rich in zinc or zinc supplementation could considerably reduce cancer risk, especially occurrence of cancers of the alimentary tract. Several studies showed significant changes in copper/zinc ratio in serum and cancer tissue between cancer patients and the general population [43–46]. In our study both increase and decrease of the Zn/Cu ratio were observed in the tested groups. Particularly large decrease of the Zn/Cu was found in the alimentary tract cancer group, most likely due to the mentioned earlier disturbances in absorption of this element in the course of alimentary tract cancers.

The results obtained in our study also showed a reduction in the concentrations of germanium and silicon in hair samples of all cancer groups. The links between these elements and the onset of cancer diseases remain to be fully elucidated. The studies on germanium carried out so far have demonstrated its potential role in the inhibition of tumor growth [47]. Recently,* in vitro* anticancer activity of organic germanium on human breast cancer cell line has been observed [48]. Aso et al. [49] showed that this activity is linked with stimulating production of gamma interferon and activation of macrophages and NK lymphocytes. The lowest germanium concentrations were found in the H and the HG groups. The question remains whether the change in germanium concentration is the result of the cancer process or whether it precedes the onset of pathological proliferation. The latter option may be supported by studies confirming the usefulness of garlic (containing high amounts of organic germanium) in cancer prevention [50].

The studies in cancer patient groups also revealed a considerable drop in silicon concentrations, which is an element belonging to the carbon group along with germanium. These results might indicate a synergism between these two elements in cancer diseases. In our study, the lowest concentration of silicon was found in the D and HG groups but without statistical significance. Silicon is found in highest amounts in the cells of connective tissue but is also an essential mineral for bone formation [51]. Moreover, its deficits in hair may result from prolonged deficiency of this element in the organism. Changes in silicon content in the connective tissue, proceeding with age, are symptomatic. Young tissues are characterized by high amounts of silicon and low amounts of calcium. Similar changes regarding these two elements are characteristic for pathologically rejuvenated cancer tissues.

Currently, potential connections between impaired glucose and insulin levels and cancer diseases are investigated. It is well known that high level of insulin is a significant risk factor for cancer [52–56]. Cancer cells are characterized by particularly high glycolytic activity, what results from lowered mitochondrial respiratory capacity in cancer tissues. Increased insulin concentration in the blood is the biochemical effect of elevated demand for glucose, which can be considered as one of the markers indicating the presence of cancer, especially leukemia [57]. This dependence is used, for example, in the PET tests [58]. Our study included patients with cancers of high glycolytic activity, such as brain tumors, lymphoma, and leukemia. The tissues of this cancer type, brain, blood, and lymphatic system, typically have very intensive glucose metabolism. Concentrations of calcium and magnesium in hair of patients from this group were the lowest as compared to the other groups. The explanation of the links between these elements and disturbed glucose metabolism in cancer tissues could be very important in order to discover their role in the etiopathogenesis of cancer diseases. Calcium has a direct effect on the energy balance of the organism. Changes in the rates of cellular ATP transport dependent on calcium concentrations can be observed in cancer cells. The other element, magnesium, serves as a catalyst for most metabolic transformations of carbohydrates. It is also known to be insulinase activator; that is, the enzyme that accelerates insulin breakdown. Low levels of magnesium are negatively correlated with the accessibility of this hormone [59].

The connection of insulin with cancer occurrence can be further confirmed by data obtained on vanadium. Due to insulin-like action of vanadium, the studies on this element confirm a connection between insulin and the onset of cancer diseases [60–62]. In our study elevated concentrations of vanadium were observed in all cancer groups.

The Ca/P ratio changed significantly in cancer groups as compared to the control group. The ratio of calcium and phosphorus determines the transfer rate of phosphate groups between high-energy compounds and has an effect on the course of energetic processes in the organism. The links between the proliferation rate and glucose metabolism depend also on the mutual ratio of sodium and potassium, Na/K. In our study, the lowest values of the Na/K ratio were found in the HG group, that is, group of cancers characterized by the highest rate of glucose metabolism.

Heavy metals and toxic elements (aluminum, lead, cadmium, mercury, and arsenic) can also be important biomarkers of cancer diseases. The data gathered in our study showed that the concentrations of aluminum and cadmium in hair were elevated in all groups of cancer patients. Within the group of hormone-dependent cancers, the concentration of these elements was lower than in the other two cancer groups, what might suggest a link between aluminum and cadmium concentration in hair and the estrogen levels in breast cancer. This type of link could be an additional biomarker of breast cancer.

Elevated cadmium levels were detected in hair of all cancer groups in relation to the control group. Moreover, a considerable decrease in the ratios of Ca/Pb and Zn/Cd, that is, ratios of the antagonists of heavy metals calcium and zinc to the heavy metals lead and cadmium, was noted in the HG and D groups. Taking into account the role of heavy metals in the etiopathogenesis of cancer, the recommendation of calcium and zinc supplementation seems justified in order to reduce cancer risk.

The results of our study allow us to conclude that hair element analysis is useful in screening tests for the biomarkers of various cancer diseases in human populations. A comparison of available results was shown in Table 4.

## 5. Conclusion

Statistical analyses indicate that levels of trace elements were statistically different in cancer groups as compared to the control group. Concentrations of 7 elements (K, Al, Hg, Ca, Na, Mo, and V) were higher and of 9 elements (B, Cu, Fe, Se, Ge, Mg, Cr, Mn, and Zn) were lower in the hormone-dependent cancer group than in the healthy group. Similarly, higher levels of 9 elements (Al, Cd, K, Hg, Na, Pb, Mo, V, and P) and lower levels of 8 elements (Mg, Ge, Se, B, Cu, Ca, Fe, and Zn) were observed in the glycolysis cancer group. Additionally, increased levels for 9 elements (Al, Hg, K, Na, V, Ca, Cd, Pb, and Mo) and decreased levels for 8 elements (Se, Zn, Mg, B, Fe, Ge, Cu, and Cr) were noted in the group with cancers of the digestive system. Lower concentrations of selenium, zinc, copper, germanium and boron, iron, and magnesium and increased level of aluminum, potassium, and molybdenum were detected in all groups of patients. Furthermore, in all cancer groups the Ca/P, Zn/Cu, and Ca/Pb ratios were changed significantly. On the basis of the obtained results it seems safe to conclude that these trace elements in hair may be regarded as tumor biomarkers and prognostic factors for various cancer groups. Moreover, our results suggest that analysis of trace element levels should be taken into consideration to optimize prevention and may be helpful to individualize therapies of various cancers in women on the basis of the analysis of hair trace elements. All in all, our results allow for the conclusion that hair element analysis is useful in screening tests for the biomarkers of various cancer diseases in female populations.

## Figures and Tables

**Table 1:** Summary of the analytical data.

Trace element	Method	Calibration curve *y* = *ax* + *b*	The linear correlation coefficient for calibration curves (*r*)	Coefficient of variance (SD_2_)	Detection limits [ppm]	Certified reference material	Sample volume
Ag	ICP/MS	a = 8302.0876 b = 502.1245	0.9996	0.00091	0.1–10	ChemLab Multi Element ICP standard (30E)	10 mL

Al	ICP/OES	a = 1022.2896 b = 29.7710	0.9997	0.001967	0.1–10	ChemLab Multi Element ICP standard (30E)	10 mL

As	ICP/MS	a = 1031.9428 b = 74.7234	0.9991	0.01291	1–100	ChemLab Multi Element ICP standard (30E)	10 mL

B	ICP/OES	a = 1056675.925 b = −103415.259	0.9992	0.000795	0.1–1	ChemLab Multi Element ICP standard (30E)	10 mL

Ba	ICP/OES	a = 84833.3333 b = −5782.3333	0.9995	0.002658	0.1–1	ChemLab Multi Element ICP standard (30E)	10 mL

Ca	ICP/OES	a = 913.2296 b = −7799.9630	0.9999	0.011560	10–100	ChemLab Multi Element ICP standard (30E)	10 mL

Cd	ICP/MS	a = 1820.5724 b = 7.2760	0.9994	0.00600	0.1–10	ChemLab Multi Element ICP standard (30E)	10 mL

Co	ICP/MS	*a* = 1077.4575 *b* = 103.9208	0.9997	0.00079	0.1–10	ChemLab Multi Element ICP standard (30E)	10 mL

Cr	ICP/MS	*a* = 8172.7995 *b* = 8239.7199	0.9999	0.00179	0.1–10	ChemLab Multi Element ICP standard (30E)	10 mL

Cu	ICP/OES	*a* = 10753.9298 *b* = −4270.2983	0.9992	0.000559	0.5–10	ChemLab Multi Element ICP standard (30E)	10 mL

Fe	ICP/OES	*a* = 2002.6296 *b* = −1813.2963	0.9995	0.004915	1–10	ChemLab Multi Element ICP standard (30E)	10 mL

Hg	ICP/MS	*a* = 8290.8350 *b* = −4100.5081	0.9998	0.00591	1–100	Merck Mercury Standard 10 mg/L Hg	10 mL

I	ICP/OES	*a* = 11245.1852 *b* = −992.5185	0.9993	0.000099	0.1–1	—	10 mL

K	ICP/OES	*a* = 58244.1053 *b* = −57032.4387	0.9994	0.003398	1–20	ChemLab Multi Element ICP standard (30E)	10 mL

Li	ICP/MS	*a* = 9890.2357 *b* = −7693.6903	0.9996	0.00597	0.1–10	ChemLab Multi Element ICP standard (30E)	10 mL

Mg	ICP/OES	*a* = 85943.4386 *b* = −33914.7193	0.9998	0.000104	0.5–10	ChemLab Multi Element ICP standard (30E)	10 mL

Mn	ICP/MS	*a* = 13218.0013 *b* = 843.5329	0.9995	0.00530	0.1–10	ChemLab Multi Element ICP standard (30E)	10 mL

Mo	ICP/MS	*a* = 4472.5926 *b* = 9.0740	0.9999	0.00179	0.1–10	ChemLab Multi Element ICP standard (30E)	10 mL

Na	ICP/OES	*a* = 43199.6140 *b* = −39009.2808	0.9991	0.004895	1–20	ChemLab Multi Element ICP standard (30E)	10 mL

Ni	ICP/MS	*a* = 2348.2015 *b* = 131.8463	0.9994	0.00091	0.1–10	ChemLab Multi Element ICP standard (30E)	10 mL

P	ICP/OES	*a* = 5153.3333 *b* = −4934.0001	0.9993	0.000159	1–20	ChemLab Phosphorus Standard solution	10 mL

Pb	ICP/MS	*a* = 7712.1212 *b* = 1929.7878	0.9997	0.00255	0.1–10	ChemLab Multi Element ICP standard (30E)	10 mL

S	ICP/OES	*a* = 447.2593 *b* = 9.0740	0.9999	0.000002	1–100	ChemLab Sulfur solution 1000 ug/mL	10 mL

Se	ICP/OES	*a* = 1124.5185 *b* = −992.5185	0.9995	0.004915	1–10	ChemLab Multi Element ICP standard (30E)	10 mL

Si	ICP/OES	*a* = 1124.5185 *b* = −992.5185	0.9991	0.003665	1–10	—	10 mL

Sn	ICP/OES	*a* = 138997.7193 *b* = −67333.5263	0.9995	0.000145	0.5-10	—	10 mL

Sr	ICP/OES	*a* = 469170.6667 *b* = −234218.666	0.9997	0.000029	0.5–1	ChemLab Multi Element ICP standard (30E)	10 mL

V	ICP/MS	*a* = 9519.6029 *b* = 1300.3729	0.9998	0.00497	0.1–10	ChemLab Multi Element ICP standard (30E)	10 mL

Zn	ICP/OES	*a* = 2024.13333 *b* = −9988.6667	0.9992	0.002791	5–10	ChemLab Multi Element ICP standard (30E)	10 mL

**Table 2:** The content of tested elements in hair of cancer patients and controls [*µ*g/g of hair].

Element	Groups			
	Control	H	HG	D
Aluminum	1.10 ± 1.01	2.23 ± 2.5^***^	3.37 ± 3.69^***^	3.97 ± 4.65^***^
Arsenic	0.02 ± 0.03	0.06 ± 0.06	0.04 ± 0.05	0.04 ± 0.06
Boron	1.05 ± 0.78	0.44 ± 0.49^***^	0.57 ± 0.62^***^	0.65 ± 0.55^***^
Barium	0.53 ± 0.44	0.70 ± 0.75	0.81 ± 0.96	0.86 ± 0.97
Cadmium	0.031 ± 0.02	0.04 ± 0.04	0.07 ± 0.07^***^	0.05 ± 0.04^***^
Calcium	425.25 ± 80.93	800.12 ± 403.05^***^	275.86 ± 102.89^∗∗∗,+++,●●●^	689.43 ± 282.36^***^
Chromium	0.39 ± 0.18	0.30 ± 0.21^***^	0.39 ± 0.33	0.38 ± 0.31^*^
Cobalt	0.03 ± 0.02	0.03 ± 0.02	0.02 ± 0.02	0.03 ± 0.02
Copper	13.15 ± 3.12	6.87 ± 2.57^∗∗∗,+++,†^	7.99 ± 4.22^***^	11.45 ± 5.67^***^
Germanium	0.04 ± 0.02	0.03 ± 0.02^***^	0.02 ± 0.01^∗∗∗,++^	0.03 ± 0.02^**^
Iodine	3.58 ± 2.15	2.92 ± 1.48	3.05 ± 1.53	3.10 ± 1.68
Iron	14.83 ± 3.06	8.83 ± 4.91^∗∗∗,++^	9.69 ± 4.84^***^	10.31 ± 4.46^***^
Lead	0.85 ± 0.54	0.94 ± 0.53	1.18 ± 0.98^*^	1.19 ± 0.91^**^
Lithium	0.03 ± 0.02	0.04 ± 0.02	0.03 ± 0.02	0.04 ± 0.04
Magnesium	27.16 ± 8.14	20.53 ± 14.06^***^	9.58 ± 4.69^∗∗∗,+++,●●●^	15.77 ± 7.61^***^
Manganese	0.86 ± 0.31	0.78 ± 0.82^**^	0.88 ± 0.70	0.95 ± 0.60
Mercury	0.02 ± 0.02	0.04 ± 0.05^***^	0.04 ± 0.06^***^	0.06 ± 0.06^***^
Molybdenum	0.03 ± 0.02	0.04 ± 0.02^***^	0.04 ± 0.02^***^	0.04 ± 0.02^***^
Nickel	1.22 ± 0.76	1.13 ± 0.86	1.25 ± 1.17	1.35 ± 0.79
Phosphorus	147.14 ± 28.38	168.18 ± 80.48	194.09 ± 78.32^∗∗∗,+++,●●^	162.35 ± 79.28
Potassium	108.97 ± 33.32	232.67 ± 188.38^***^	243.83 ± 193.92^***^	221.46 ± 155.48^***^
Selenium	0.42 ± 0.12	0.26 ± 0.14^***^	0.22 ± 0.12^***^	0.21 ± 0.12^∗∗∗,●●^
Silicon	40.05 ± 10.97	11.84 ± 14.21	12.99 ± 13.89	14.20 ± 11.56
Sodium	229.76 ± 50.08	365.77 ± 222.04^***^	361.89 ± 216.12^***^	403.08 ± 234.42^***^
Strontium	1.89 ± 1.17	2.17 ± 1.25	2.57 ± 1.36	1.89 ± 1.18
Sulfur	24916 ± 8973	22496 ± 8215	21060 ± 4256	25962 ± 27886
Tin	0.04 ± 0.03	0.07 ± 0.05	0.05 ± 0.06	0.07 ± 0.07
Vanadium	0.03 ± 0.02	0.04 ± 0.02^**^	0.04 ± 0.02^***^	0.05 ± 0.03^***^
Zinc	141.23 ± 32.11	130.58 ± 39.01^∗,+++^	125.09 ± 47.28^**^	74.55 ± 27.53^∗∗∗,†††,●●●^

H, HG, and D, respectively, are hormone dependent cancers, cancers with high glycolytic activity, and alimentary tract cancers; values expressed as mean ± SEM.

^∗∗∗,∗∗,∗^Statistical difference versus control, *P* < 0.001, *P* < 0.01, *P* < 0.05, respectively.

^+++,++,+^Statistical difference versus D group, *P* < 0.001, *P* < 0.01, *P* < 0.05, respectively.

^†††,††,†^Statistical difference versus HG group, *P* < 0.001, *P* < 0.01, *P* < 0.05, respectively.

^●●●,●●,●^Statistical difference versus H group, *P* < 0.001, *P* < 0.01, *P* < 0.05, respectively.

**Table 3:** The ratio of concentrations of elements in hair of cancer patients and controls.

Ratio of elements concentration	Groups			
	Control	H	HG	D
Ca/P	2.92 ± 0.41	5.78 ± 5.55^***^	1.50 ± 0.43^∗∗∗,+++,●●●^	4.72 ± 2.29^***^
Na/K	2.21 ± 0.69	2.03 ± 1.16^***^	1.95 ± 1.05^**^	2.11 ± 1.06
Ca/K	4.19 ± 1.37	6.91 ± 7.68	1.96 ± 1.69^***^	4.97 ± 4.23
Zn/Cu	11.18 ± 3.57	22.25 ± 12.62^∗∗∗,+++,††^	17.62 ± 8.79^***^	7.79 ± 4.64^***^
Na/Mg	9.06 ± 3.70	27.27 ± 28.82	55.89 ± 74.94	32.85 ± 28.87
Ca/Mg	16.56 ± 5.30	49.03 ± 27.87^***^	36.62 ± 22.51	52.55 ± 31.78^∗∗∗,†††^
Fe/Cu	1.18 ± 0.36	1.58 ± 1.26	1.45 ± 1.18	1.07 ± 0.67
Ca/Na	1.92 ± 0.5167	3.68 ± 3.86^*^	1.11 ± 0.85^***^	2.64 ± 2.34
Cu/Mo	4567.97 ± 38.69	208.44 ± 117.66	294.89 ± 447.84	363.49 ± 359.24
Fe/Co	880.31 ± 733.08	619.13 ± 1096.63	753.38 ± 1133.30	1215.35 ± 3115.88
Ca/Sr	322.75 ± 206.99	892.70 ± 2970.08	209.99 ± 563.70	1123.10 ± 2343.98
Ca/Fe	29.97 ± 8.65	119.44 ± 85.89	34.28 ± 17.39	82.89 ± 52.03
Ca/Pb	1987.95 ± 11736.03	1381.08 ± 2082.62^***^	439.00 ± 595.72^***^	1157.92 ± 1810.36^*^
Zn/Cd	7328 ± 7941	13185 ± 23996	5165 ± 11178^***^	4526 ± 8822^***^
Fe/Pb	81.23 ± 539.11	11.91 ± 10.46	13.43 ± 16.09	15.59 ± 21.79
K/Li	8489 ± 18189	13331 ± 38908	24862 ± 119467	12643 ± 26360
K/Co	6736 ± 7749	14980 ± 18184	22624 ± 39103	25265 ± 59991
Ca/Si	12.62 ± 10.07	994.61 ± 5389.21	125.39 ± 354.45	274.26 ± 1503.35
I/Se	8.94 ± 5.70	15.49 ± 12.86	61.27 ± 191.47	67.59 ± 277.01
Mg/Pb	140.95 ± 794.29	34.50 ± 44.93	18.45 ± 27.11	26.71 ± 42.56

H, HG, and D, respectively, are hormone dependent cancers, cancers with high glycolytic activity, and alimentary tract cancers; values expressed as mean ± SEM.

^∗∗∗,∗∗,∗^Statistical difference versus control, *P* < 0.001, *P* < 0.01, *P* < 0.05, respectively.

^+++,++,+^Statistical difference versus D group, *P* < 0.001, *P* < 0.01, *P* < 0.05, respectively.

^†††,††,†^Statistical difference versus HG group, *P* < 0.001, *P* < 0.01, *P* < 0.05, respectively.

^●●●,●●,●^Statistical difference versus H group, *P* < 0.001, *P* < 0.01, *P* < 0.05, respectively.

**Table 4:** Comparison of available studies in concentrations of trace elements in scalp hair samples of different types of cancer patients and healthy participants.

Reference	Concentrations (mean ± SD)		Type of cancer	Method	Age	*N* (healthy + cancer patients)	Sex	Country
	Healthy participants	Cancer patients						
Al (Aluminum)								
Cihan et al., 2011 [26] [*µ*g/g]	15.245 ± 15.909	21.017 ± 17.556	Breast cancer (stage III)	ICP/MS	25–65	52 + 52	F	Turkey
Benderli Cihan and Öztürk Yildirim, 2011 [16] [*µ*g/kg]	11.366 ± 12.685	16.460 ± 16.313	Non-small cell lung cancer	ICP/MS	42–77	74 + 67	M	Turkey

As (Arsenic)								
Cihan et al., 2011 [26] [*µ*g/g]	0.239 ± 0.220	1.522 ± 1.980	Breast cancer (stage III)	ICP/MS	25–65	52 + 52	F	Turkey
Benderli Cihan and Öztürk Yildirim, 2011 [16] [*µ*g/kg]	0.558 ± 0.742	0.458 ± 1.269	Non-small cell lung cancer	ICP/MS	42–77	74 + 67	M	Turkey
Wadhwa et al., 2015 [27] [*µ*g/g]	0.58 ± 0.14	2.94 ± 0.56	Breast cancer	AAS	n.i.	94 + 47	F	Pakistan
	0.58 ± 0.14	3.05 ± 0.75	Cervix cancer	AAS	n.i.	94 + 31	F	Pakistan
	0.58 ± 0.14	2.71 ± 0.32	Ovarian cancer	AAS	n.i.	94 + 19	F	Pakistan
	0.58 ± 0.14	3.55 ± 0.64	Mouth cancer	AAS	n.i.	94 + 28	F	Pakistan

B (Boron)								
Cihan et al., 2011 [26] [*µ*g/g]	0.345 ± 0.387	0.704 ± 0.567	Breast cancer (stage III)	ICP/MS	25–65	52 + 52	F	Turkey
Benderli Cihan and Öztürk Yildirim, 2011 [16] [*µ*g/kg]	1.136 ± 1.915	1.461 ± 1.972	Non-small cell lung cancer	ICP/MS	42–77	74 + 67	M	Turkey

Ba (Barium)								
Cihan et al., 2011 [26] [*µ*g/g]	1.763 ± 1.848	1.488 ± 0.938	Breast cancer (stage III)	ICP/MS	25–65	52 + 52	F	Turkey
Benderli Cihan and Öztürk Yildirim, 2011 [16] [*µ*g/kg]	1.396 ± 1.513	1.461 ± 1.972	Non-small cell lung cancer	ICP/MS	42–77	74 + 67	M	Turkey

Ca (Calcium)								
Cihan et al., 2011 [26] [*µ*g/g]	34.372 ± 19.567	25.849 ± 7.877	Breast cancer (stage III)	ICP/MS	25–65	52 + 52	F	Turkey
Benderli Cihan and Öztürk Yildirim, 2011 [16] [*µ*g/kg]	30.812 ± 18.809	68.250 ± 61.327	Non-small cell lung cancer	ICP/MS	42–77	74 + 67	M	Turkey

Cd (Cadmium)								
Cihan et al., 2011 [26] [*µ*g/g]	0.175 ± 0.206	0.441 ± 0.486	Breast cancer (stage III)	ICP/MS	25–65	52 + 52	F	Turkey
Benderli Cihan and Öztürk Yildirim, 2011 [16] [*µ*g/kg]	0.316 ± 0.426	0.209 ± 0.176	Non-small cell lung cancer	ICP/MS	42–77	74 + 67	M	Turkey
Wadhwa et al., 2015 [27] [*µ*g/g]	1.4 ± 0.56	4.27 ± 1.58	Breast cancer	AAS	n.i.	94 + 47	F	Pakistan
	1.4 ± 0.56	5.75 ± 0.96	Cervix cancer	AAS	n.i.	94 + 31	F	Pakistan
	1.4 ± 0.56	5.34 ± 1.45	Ovarian cancer	AAS	n.i.	94 + 19	F	Pakistan
	1.4 ± 0.56	6.73 ± 1.65	Mouth cancer	AAS	n.i.	94 + 28	F	Pakistan
Pasha et al., 2010 [28] [*µ*g/g]	1.675 ± 1.127	16.82 ± 13.62	Gastrointestinal cancer	AAS	37–65	37 + 36	F + M	Pakistan

Co (Cobalt)								
Cihan et al., 2011 [26] [*µ*g/g]	0.269 ± 0.390	0.664 ± 0.566	Breast cancer (stage III)	ICP/MS	25–65	52 + 52	F	Turkey
Benderli Cihan and Öztürk Yildirim, 2011 [16] [*µ*g/kg]	0.392 ± 0.467	3.131 ± 11.057	Non-small cell lung cancer	ICP/MS	42–77	74 + 67	M	Turkey

Cr (Chromium)								
Cihan et al., 2011 [26] [*µ*g/g]	0.991 ± 0.950	1.253 ± 0.891	Breast cancer (stage III)	ICP/MS	25–65	52 + 52	F	Turkey
Benderli Cihan and Öztürk Yildirim, 2011 [16] [*µ*g/kg]	0.934 ± 1.016	2.492 ± 4.021	Non-small cell lung cancer	ICP/MS	42–77	74 + 67	M	Turkey
Pasha et al., 2010 [28] [*µ*g/g]	2.345 ± 1.083	2.509 ± 2.080	Gastrointestinal cancer	AAS	37–65	37 + 36	F + M	Pakistan

Cu (Copper)								
Cihan et al., 2011 [26] [*µ*g/g]	11.932 ± 10.435	13.546 ± 18.719	Breast cancer (stage III)	ICP/MS	25–65	52 + 52	F	Turkey
Benderli Cihan and Öztürk Yildirim, 2011 [16] [*µ*g/kg]	15.753 ± 16.727	24.453 ± 18.465	Non-small cell lung cancer	ICP/MS	42–77	74 + 67	M	Turkey
Ahmad et al., 2011 [29] [*µ*g/mL]	18.00 ± 0.08	21.64 ± 1.63	Lymphoma	AAS	18–80	n.i.	F + M	Pakistan
Khuder et al., 2014 [30] [*µ*g/g]	15.5	12.1	Leukaemia	XRF	16–93	39 + 39	M	Syria
Ahmad et al., 2011 [29] [*µ*g/mL]	18.03 ± 0.55	27.30 ± 0.95	Esophageal cancer	AAS	18–80	n.i.	F + M	Pakistan
Pasha et al., 2010 [28] [*µ*g/g]	21.08 ± 5.726	30.20 ± 19.03	Gastrointestinal cancer	AAS	37–65	37 + 36	F + M	Pakistan

Fe (Iron)								
Cihan et al., 2011 [26] [*µ*g/g]	28.190 ± 23.865	32.883 ± 35.260	Breast cancer (stage III)	ICP/MS	25–65	52 + 52	F	Turkey
Benderli Cihan and Öztürk Yildirim, 2011 [16] [*µ*g/kg]	25.052 ± 22.929	9.652 ± 6.643	Non-small cell lung cancer	ICP/MS	42–77	74 + 67	M	Turkey
Ahmad et al., 2011 [29] [*µ*g/mL]	22.85 ± 1.30	120.34 ± 3.41	Lymphoma	AAS	18–80	n.i.	F + M	Pakistan
Khuder et al., 2014 [30] [*µ*g/g]	35.2	38.4	Leukaemia	XRF	16–93	39 + 39	M	Syria
Ahmad et al., 2011 [29] [*µ*g/mL]	22.82 ± 1.81	22.07 ± 1.70	Esophageal cancer	AAS	18–80	n.i.	F + M	Pakistan
Pasha et al., 2010 [28] [*µ*g/g]	66.35 ± 40.66	117.2 ± 77.90	Gastrointestinal cancer	AAS	37–65	37 + 36	F + M	Pakistan

Hg (Mercury)								
Cihan et al., 2011 [26] [*µ*g/g]	0.652 ± 0.596	0.473 ± 0.604	Breast cancer (stage III)	ICP/MS	25–65	52 + 52	F	Turkey
Benderli Cihan and Öztürk Yildirim, 2011 [16] [*µ*g/kg]	0.585 ± 0.713	1.233 ± 1.367	Non-small cell lung cancer	ICP/MS	42–77	74 + 67	M	Turkey

K (Potassium)								
Cihan et al., 2011 [26] [*µ*g/g]	11.009 ± 8.226	13.231 ± 11.671	Breast cancer (stage III)	ICP/MS	25–65	52 + 52	F	Turkey
Benderli Cihan and Öztürk Yildirim, 2011 [16] [*µ*g/kg]	10.701 ± 9.252	15.322 ± 18.655	Non-small cell lung cancer	ICP/MS	42–77	74 + 67	M	Turkey

Li (Lithium)								
Cihan et al., 2011 [26] [*µ*g/g]	0.503 ± 0.656	0.912 ± 0.615	Breast cancer (stage III)	ICP/MS	25–65	52 + 52	F	Turkey
Benderli Cihan and Öztürk Yildirim, 2011 [16] [*µ*g/kg]	0.571 ± 0.586	0.595 ± 0.670	Non-small cell lung cancer	ICP/MS	42–77	74 + 67	M	Turkey

Mg (Magnesium)								
Cihan et al., 2011 [26] [*µ*g/g]	41.005 ± 20.748	31.276 ± 16.873	Breast cancer (stage III)	ICP/MS	25–65	52 + 52	F	Turkey
Benderli Cihan and Öztürk Yildirim, 2011 [16] [*µ*g/kg]	31.921 ± 21.315	28.920 ± 24.203	Non-small cell lung cancer	ICP/MS	42–77	74 + 67	M	Turkey
Ahmad et al., 2011 [29] [*µ*g/mL]	120.34 ± 3.4	67.46 ± 22.02	Lymphoma	AAS	18–80	n.i.	F + M	Pakistan
	120.98 ± 10.32	74.25 ± 10.01	Esophageal cancer	AAS	18–80	n.i.	F + M	Pakistan

Mn (Manganese)								
Cihan et al., 2011 [26] [*µ*g/g]	0.863 ± 0.675	0.989 ± 0.924	Breast cancer (stage III)	ICP/MS	25–65	52 + 52	F	Turkey
Benderli Cihan and Öztürk Yildirim, 2011 [16] [*µ*g/kg]	1.144 ± 1.119	1.8193 ± 2.160	Non-small cell lung cancer	ICP/MS	42–77	74 + 67	M	Turkey

Na (Sodium)								
Cihan et al., 2011 [26] [*µ*g/g]	28.641 ± 21.895	29.666 ± 19.391	Breast cancer (stage III)	ICP/MS	25–65	52 + 52	F	Turkey
Benderli Cihan and Öztürk Yildirim, 2011 [16] [*µ*g/kg]	21.378 ± 15.798	23.316 ± 30.458	Non-small cell lung cancer	ICP/MS	42–77	74 + 67	M	Turkey

Ni (Nickel)								
Cihan et al., 2011 [26] [*µ*g/g]	0.688 ± 0.731	0.571 ± 0.499	Breast cancer (stage III)	ICP/MS	25–65	52 + 52	F	Turkey
Benderli Cihan and Öztürk Yildirim, 2011 [16] [*µ*g/kg]	0.580 ± 0.547	1.126 ± 0.800	Non-small cell lung cancer	ICP/MS	42–77	74 + 67	M	Turkey
Ahmad et al., 2011 [29] [*µ*g/mL]	5.37 ± 0.23	5.92 ± 0.58	Lymphoma	AAS	18–80	n.i.	F + M	Pakistan
Khuder et al., 2014 [30] [*µ*g/g]	1.30	2.32	Leukaemia	XRF	16–93	39 + 39	M	Syria
Ahmad et al., 2011 [29] [*µ*g/mL]	5.40 ± 0.45	9.85 ± 0.60	Esophageal cancer	AAS	18–80	n.i.	F + M	Pakistan
Wadhwa et al. 2015 [27] [*µ*g/g]	0.63 ± 0.16	1.51 ± 0.37	Breast cancer	AAS	n.i.	94 + 47	F	Pakistan
	0.63 ± 0.16	1.89 ± 0.40	Cervix cancer	AAS	n.i.	94 + 31	F	Pakistan
	0.63 ± 0.16	1.25 ± 0.51	Ovarian cancer	AAS	n.i.	94 + 19	F	Pakistan
	0.63 ± 0.16	2.92 ± 0.57	Mouth cancer	AAS	n.i.	94 + 28	F	Pakistan
Pasha et al., 2010 [28] [*µ*g/g]	4.309 ± 2.631	5.971 ± 4.622	Gastrointestinal cancer	AAS	37–65	37 + 36	F + M	Pakistan

Pb (Lead)								
Cihan et al., 2011 [26] [*µ*g/g]	6.196 ± 7.491	3.794 ± 3.292	Breast cancer (stage III)	ICP/MS	25–65	52 + 52	F	Turkey
Benderli Cihan and Öztürk Yildirim, 2011 [16] [*µ*g/kg]	5.090 ± 6.198	8.577 ± 19.876	Non-small cell lung cancer	ICP/MS	42–77	74 + 67	M	Turkey
Ahmad et al., 2011 [29] [*µ*g/mL]	15.91 ± 0.73	26.31 ± 2.33	Lymphoma	AAS	18–80	n.i.	F + M	Pakistan
Khuder et al., 2014 [30] [*µ*g/g]	10.9	8.28	Leukaemia	XRF	16–93	39 + 39	M	Syria
Ahmad et al., 2011 [29] [*µ*g/mL]	15.91 ± 0.99	26.70 ± 5.97	Esophageal cancer	AAS	18–80	n.i.	F + M	Pakistan
Pasha et al., 2010 [28] [*µ*g/g]	15.50 ± 8.119	28.71 ± 16.85	Gastrointestinal cancer	AAS	37–65	37 + 36	F + M	Pakistan

Se (Selenium)								
Cihan et al., 2011 [26] [*µ*g/g]	5.061 ± 7.597	0.649 ± 0.930	Breast cancer (stage III)	ICP/MS	25–65	52 + 52	F	Turkey
Benderli Cihan and Öztürk Yildirim, 2011 [16] [*µ*g/kg]	20.135 ± 21.042	13.703 ± 19.784	Non-small cell lung cancer	ICP/MS	42–77	74 + 67	M	Turkey

Sr (Strontium)								
Cihan et al., 2011 [26] [*µ*g/g]	0.499 ± 0.502	0.449 ± 0.419	Breast cancer (stage III)	ICP/MS	25–65	52 + 52	F	Turkey
Benderli Cihan and Öztürk Yildirim, 2011 [16] [*µ*g/kg]	1.455 ± 1.678	1.910 ± 1.914	Non-small cell lung cancer	ICP/MS	42–77	74 + 67	M	Turkey

Ti (Tin)								
Cihan et al., 2011 [26] [*µ*g/g]	0.708 ± 0.510	0.653 ± 0.465	Breast cancer (stage III)	ICP/MS	25–65	52 + 52	F	Turkey
Benderli Cihan and Öztürk Yildirim, 2011 [16] [*µ*g/kg]	2.079 ± 4.464	12.638 ± 19.128	Non-small cell lung cancer	ICP/MS	42–77	74 + 67	M	Turkey

V (Vanadium)								
Cihan et al., 2011 [26] [*µ*g/g]	0.475 ± 0.439	0.640 ± 0.752	Breast cancer (stage III)	ICP/MS	25–65	52 + 52	F	Turkey
Benderli Cihan and Öztürk Yildirim, 2011 [16] [*µ*g/kg]	1.047 ± 1.742	1.835 ± 2.290	Non-small cell lung cancer	ICP/MS	42–77	74 + 67	M	Turkey

Zn (Zinc)								
Cihan et al., 2011 [26] [*µ*g/g]	63.700 ± 62.183	29.374 ± 20.367	Breast cancer (stage III)	ICP/MS	25–65	52 + 52	F	Turkey
Benderli Cihan and Öztürk Yildirim, 2011 [16] [*µ*g/kg]	109.763 ± 95.334	53.22 ± 60.301	Non-small cell lung cancer	ICP/MS	42–77	74 + 67	M	Turkey
Ahmad et al., 2011 [29] [*µ*g/mL]	174.99 ± 9.57	167.80 ± 13.86	Lymphoma	AAS	18–80	n.i.	F + M	Pakistan
Khuder et al., 2014 [30] [*µ*g/g]	218	183	Leukaemia	XRF	16–93	39 + 39	M	Syria
Ahmad et al., 2011 [29] [*µ*g/mL]	175.32 ± 11.48	144.85 ± 11.33	Esophageal cancer	AAS	18–80	n.i.	F + M	Pakistan
Wadhwa et al., 2015 [27] [*µ*g/g]	247 ± 17.4	114 ± 14.0	Breast cancer	AAS	n.i.	94 + 47	F	Pakistan
	247 ± 17.4	168 ± 31.0	Cervix cancer	AAS	n.i.	94 + 31	F	Pakistan
	247 ± 17.4	125 ± 7.63	Ovarian cancer	AAS	n.i.	94 + 19	F	Pakistan
	247 ± 17.4	161 ± 24.6	Mouth cancer	AAS	n.i.	94 + 28	F	Pakistan
Pasha et al., 2010 [28] [*µ*g/g]	140.7 ± 79.48	370.5 ± 208.1	Gastrointestinal cancer	AAS	37–65	37 + 36	F + M	Pakistan
Memon et al., 2007 [21] [*μ*g/g]	245.6 ± 15.6	125.5 ± 11.5	Ovarian cancer	FAAS	30–60	50 + 35	F	Pakistan
	245.6 ± 15.6	114.5 ± 12.9	Breast cancer	FAAS	30–60	50 + 30	F	Pakistan

Abbreviations: AAS—atomic absorption spectroscopy; FAAS—flame atomic absorption spectrometry; XRF—X-ray fluorescence; n.i.—no information.
